# Cisplatin-resistant HepG2 cell-derived exosomes transfer cisplatin resistance to cisplatin-sensitive cells in HCC

**DOI:** 10.7717/peerj.11200

**Published:** 2021-04-13

**Authors:** Zuxiong Tang, Jun He, Jiayue Zou, Shufei Yu, Xiaoming Sun, Lei Qin

**Affiliations:** 1 Department of Hepato-Pancreatico-Biliary Surgery, The First Affiliated Hospital of Soochow University, Suzhou, Jiangsu, China; 2 Department of General Surgery, Suzhou Wuzhong People’s Hospital, Suzhou, Jiangsu, China

**Keywords:** Drug resistance, Cell viability

## Abstract

**Backgrounds:**

Cancer cell resistance to chemotherapy drugs such as Gemcitabine, Oxaliplatin, Cisplatin, Doxorubicin, and 5-fluorouracil account for the main reason of chemotherapy failure for HCC patients, especially for those with advanced HCC or metastasis patients. This emerging resistance limits the effectiveness and clinical application of these chemotherapy drugs. Previous studies reported that drug-resistant tumor cell-derived exosomes could transfer their resistance property to tumor sensitive cells in some cancer, including lung and gastric cancer. This study sought to explore whether HepG2/DDP cell-derived exosomes transmit cisplatin (DDP) resistance to HepG2 and other HCC sensitive cells, and provide considerable guidance for HCC nursing with Cisplatin DDP clinically.

**Methods:**

The HepG2 DDP-resistant cell line (HepG2/DDP) was established, and the exosomes from both HepG2/DDP and HepG2 cells were isolated and named ES-2, ES-1, respectively. HepG2 or SMMC-7721 or Huh7 cells were treated with DDP or DDP + ES-2, and HepG2/DDP cells were treated with ES-1. Then, the activation of drug resistance via HepG2/DDP exosomes transfer to HepG2, SMMC-7721 and Huh7 cells were assessed by cell viability assay and ROS formation. Moreover, the relative expression of P-glycoprotein (P-gp) was measured by western blot analysis.

**Results:**

HepG2/DDP cell-derived exosomes were successfully isolated from cisplatin-resistant HepG2 cells, and named ES-2. Cell viability of HepG2 or SMMC-7721 or Huh7 cells treated with DDP + ES-2 was enhanced compared with that of DDP treatment group. Also, the concentration of ROS generated in cells under DDP or DDP + ES-2 treatment was strongly increased compared with that of control, although the concentration of ROS was clearly smaller in DDP + ES-2 treatment group compared with DDP treatment. At the same time, the expression of P-gp was upregulated on the ES-2 surface.

**Conclusion:**

The results mentioned above clarified that HepG2/DDP cell-derived exosomes conferred cisplatin resistance to HepG2 and other HCC cell lines, and provided a new significance for improving the effectiveness of DDP in treating HCC.

## Introduction

Hepatocellular carcinoma (HCC) is ranked the fifth pervasive malignant tumor; it is also one of the top lethal causes among human cancers (Torre et al., 2015). Because of the scarcity of clinical symptoms in the early stage of HCC, many patients are diagnosed with advanced HCC, stage of metastasis in certain cases, and poor hepatic reserve in others (Kalyan, Nimeiri & Kulik, 2015) Chemotherapy with a first-line anti-tumor drugs, such as adriamycin, 5-fluorouracil and cisplatin have improved the survival rate among these patients over the last decades, except for surgical resection. However, emerging chemotherapy drug resistances to these pivotal therapeutic agents also resulted in a lower 5-year survival rate of patients with HCC (Llovet et al., 2015; El-Serag, 2011).

Exosomes are a kind of nano-sized extracellular microvesicles (40–100 nm in diameter) secreted by many cell types, including T cells, B cells, dendritic cells, epithelial cells, and tumor cells (Zhang et al., 2015; Wahlgren et al., 2012). Exosomes are widely distributed in various human body fluids. They play an important role in cell-to-cell communication and regulate the cellular microenvironment through several biological molecules, including DNA, RNA and proteins (Paladini et al., 2016). Recent studies have provided a growing number of literature showing different influences of tumor cells-derived exosomes on tumor progression, metastasis, chemoresistance and immune dysregulation (Martins, Dias & Hainaut, 2013). Among them, more attention was addressed to the role of tumor cell-derived exosomes in chemoresistance (Sharma, 2017; Butera, Pacchiana & Donadelli, 2018). The DDP (Diamminedichloroplatinum) or Cisplatin is a chemotherapy medication utilized as a treatment for many types of cancers, addicted by injection into patient veins. In 2017, Qin et al. (2017) reported that DDP-resistant A549-derived exosomes can alter other lung cancer cells’ sensitivity to DDP in exosomal miR-100–5p-dependent manner in lung cancer. Meanwhile, another study revealed that HCC cell-derived exosomes induced sorafenib resistance in hepatocellular carcinoma both in vivo and in vitro (Qu et al., 2016). Previous reports suggested the resistance of tumor cells to drugs could produce a plethora of exosomes that may transfer genetic information to other tumor cells (Corcoran et al., 2012; Safaei et al., 2005).

However, P-glycoprotein (P-gp) drug is reputed to grant multidrug tolerance in various cancer chemotherapy. The P-gp was found to be strongly expressed in several types of cancer cells as well as some normal tissues like the epithelial cells of the intestine and the endothelial cells in the brain. In addition, a recent study analyzed the impact of P-gp in both immune cells and cancer cells. The authors highlighted its role in the tumor microenvironment by using P-gp inhibitors against anti-cancer therapy. Therefore, regarding its location and protein expression, P-gp was proposed as having a crucial function in the brain uptake, intestinal absorption mechanism of the intestine, and cancer chemotherapy (Lin & Yamazaki, 2003).

In this study, we evaluated whether exosomes from HepG2/DDP cells transmit chemoresistance to DDP-sensitive HepG2 cells and other HCC cell lines. In this vein, we manufactured a cisplatin resistance HCC cell line HepG2 (HepG2/DDP), and exosomes were isolated from HepG2 and HepG2/DDP cells. Afterward, the exosomes derived from HepG2/DDP cells were used to treat HepG2 or other HCC cells. Then, the cell viability and the concentration of intracellular ROS formation of both treated and untreated cells were assessed and compared. Furthermore, protein expressions of P-gp from DDP-sensitive or DDP-resistant HepG2 cell-derived exosomes were measured, respectively. These findings indicated that exosomes from HepG2/DDP cell transferred DDP-resistance to DDP-sensitive cells, which may provide a new significance for improving the effectiveness of DDP in treating HCC.

## Materials and Methods

### Cell lines and cell culture

Three hepatocellular carcinoma cell lines named HepG2, SMMC-7721 and Huh7 were purchased from American Type Culture Collection (ATCC, USA). Among them, HepG2 and SMMC-7721 were cultured in RPMI-1640 medium (Nanjing Kaiji Biology, China) and Huh7 was cultivated in DMEM medium (Nanjing Kaiji Biology, China), both containing 10% FBS (fetal bovine serum, Nanjing Kaiji Biology, China) at the atmosphere of 37 °C and 5% CO_2_.

### Establishment of cisplatin-resistant HepG2 (HepG2/DDP)

HepG2 cells were exposed to cisplatin (DDP, Sigma-Aldrich, Germany) at the concentration of 0.1 mM, and followed by a sub-culture in the RPMI-1640 medium containing stepwise-increasing concentrations of DDP (0.1, 0.5, 1.0, 2.0 and 3.0 mM), timely. Finally, viable HepG2 cells in the culture medium with a high concentration of DDP (2.0 mM) were selected and defined as cisplatin-resistant HepG2 cells (HepG2/DDP).

### Extraction of exosomes from HepG2/DDP and HepG2 cells

HepG2 and HepG2 resistance to DDP (HepG2/DDP) were cultured with medium under the same condition of non-FBS for 48 h. Exosome from eight mL cellular supernatant was isolated by exoEasy Maxi Kit (QIAGEN, Germany) according to the manufacture’s instruction. Then, exosomal proteins were quantified by BCA protein assay kit (Beyotime Biotechnology, Nantong, China) and the exosome markers, including CD9, CD63 and CD81, were measured by western blot analysis.

### Co-culture assay

Exosomes from HepG2 and HepG2/DDP were named ES-1 and ES-2, respectively. For HepG2/DDP cells, there were two groups, including control (no treatment) and ES-1 treatment group. The HCC sensitive cells, including HepG2, SMMC-7721 and Huh7 divided into three groups, including control (vehicle treatment), DDP treatment and DDP + ES-2 treatment. Total exosomes isolated from eight mL cellular supernatant at a dilution of 100 times were used to treat cells, and cells were harvested for further experiment after 24 h.

### Cell viability assay

MTT assay was conducted to measure cell cytotoxicity of DDP and cell proliferation. Cells in the logarithmic growth phase ( 1 ×10^4^ cells/mL) were plated in 96-well plates. For cells’ cytotoxicity assay, cells were treated with different concentrations of DDP for 4 h. MTT (5 mg/mL) was added to cells and the absorbance was measured at 570 nm. The cell growth inhibition curve was drawn and the inhibitory concentration to produce 50% cell death (IC50) of DDP was calculated.

For HCC cell proliferation, cells were treated with HepG2-exosome or HepG2/DDP-exosome for 4 h. Then, 200 µL MTT (5 mg/mL) was added to the treated cells and the absorbance was also measured at 570 nm. The inhibition rate of cell growth was calculated using the following equation percent survival = (1-ODexperiment/ODcontrol) ×100%.

### Measurement of reactive oxygen species (ROS)

The formation of intracellular ROS was quantified using a ROS assay kit (DCFH-DA) by SpectraMax iD5 (Molecular Devices, USA). Briefly, DCFH-DA was diluted with the corresponding culture medium and added to cells for incubation. After incubation for 3 h, the cell supernatant was separated from cells and the fluorescence was assessed using a fluorometer at the excitation wavelength of 500 nm and an emission wavelength of 530 nm.

### Western blot analysis

The total proteins were extracted from cells, and the protein concentration was quantified using a BCA kit. The equal amount of protein was loaded and separated on a 10% SDS-PAGE gel and was transferred onto the PVDF membrane. Skim milk powder in PBST (5%, w/v) was used to blocking for 2 h at 22−25 °C. The PVDF membrane was incubated with a primary antibody for 1 h at 22−25 °C, and then was washed 3 times with PBST for 10 min each time, including CD9 (Abcam, ab92726), CD63 (Abcam, ab217345), CD81 (Abcam, ab79559), P-gp (Santa Cruz Biotechnology, sc-13131), LAMP2 (Santa Cruz Biotechnology,sc-18822) and GAPDH (Santa Cruz Biotechnology, sc-47724). The PVDF membrane was incubated for another 1 h with the corresponding secondary antibody and ECL substrate (ECL; Thermo Scientific, Rockford, IL, USA) was then added after washing, followed by visualization using film exposure.

### Statistical analysis

All the experiment values were displayed as average ± standard deviation (SD). ANOVA for *t*-test was implemented using SPSS version 20.0. After analysis, *P* < 0.05 (two-sided) was considered statistically significant.

## Results

### Characterization of isolated exosomes

**Figure 1:**
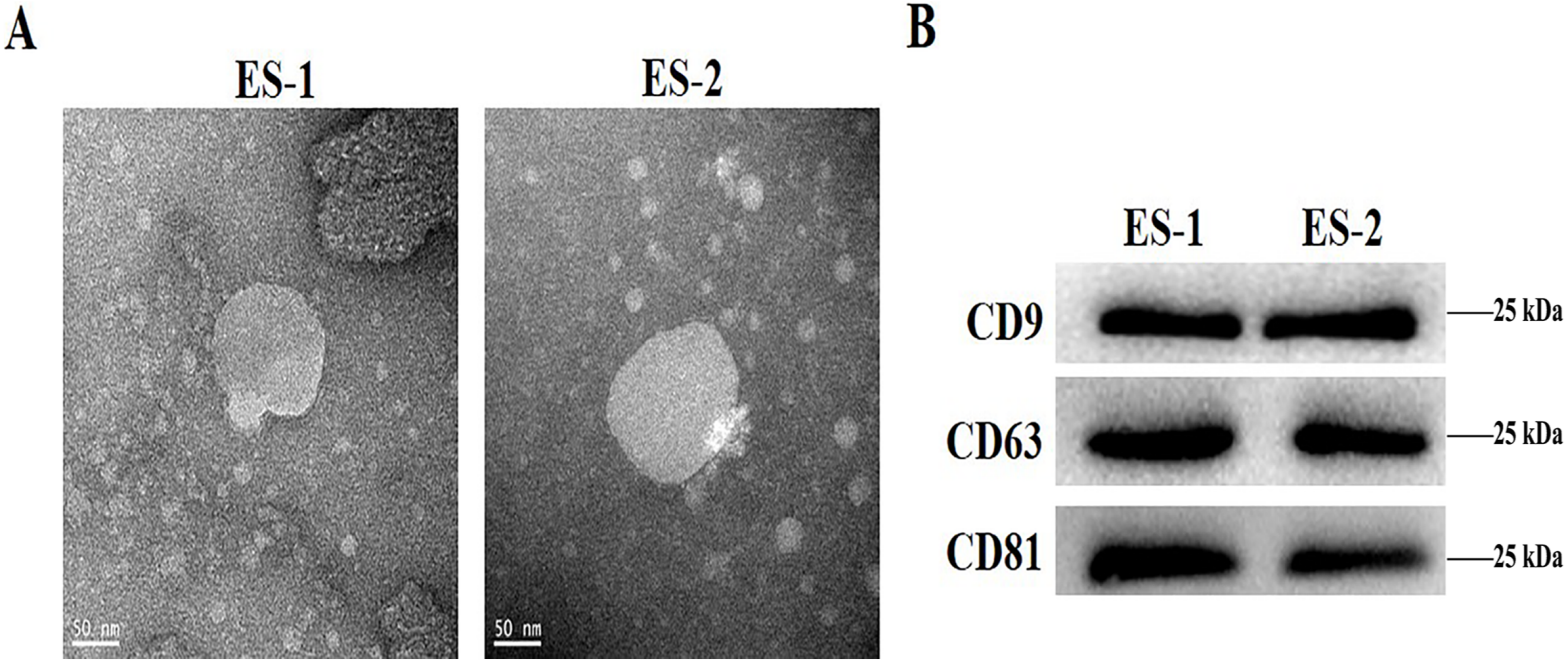
Characterization of isolated exosomes. (A) Representative transmission electron microscope image of exosomes from HepG2 cells and HepG2 DDP-resistant cells, named ES-1 and ES-2, respectively (scale bar, 50 nm). (B) Western blot showed that CD9, CD63 and CD81 were expressed in ES-1 and ES-2.

To determine the effects of exosomes isolated from cisplatin-resistant cell, exosomes were isolated from HepG2 and HepG2/DDP cells, and named ES-1 and ES-2, respectively. Observations through the transmission electron microscope showed that the two exosomes were typical round vesicles (Fig. 1A). Meanwhile, results from the western blot analysis showed the expression of the three exosome markers (CD9, CD63 and CD81) in these two exosomes (Fig. 1B). This indicates that the isolation of exosomes was well-conducted and successful.

### HepG2/DDP cell-derived exosomes affected cell viability and ROS formation in HepG2 cells in vitro

**Figure 2:**
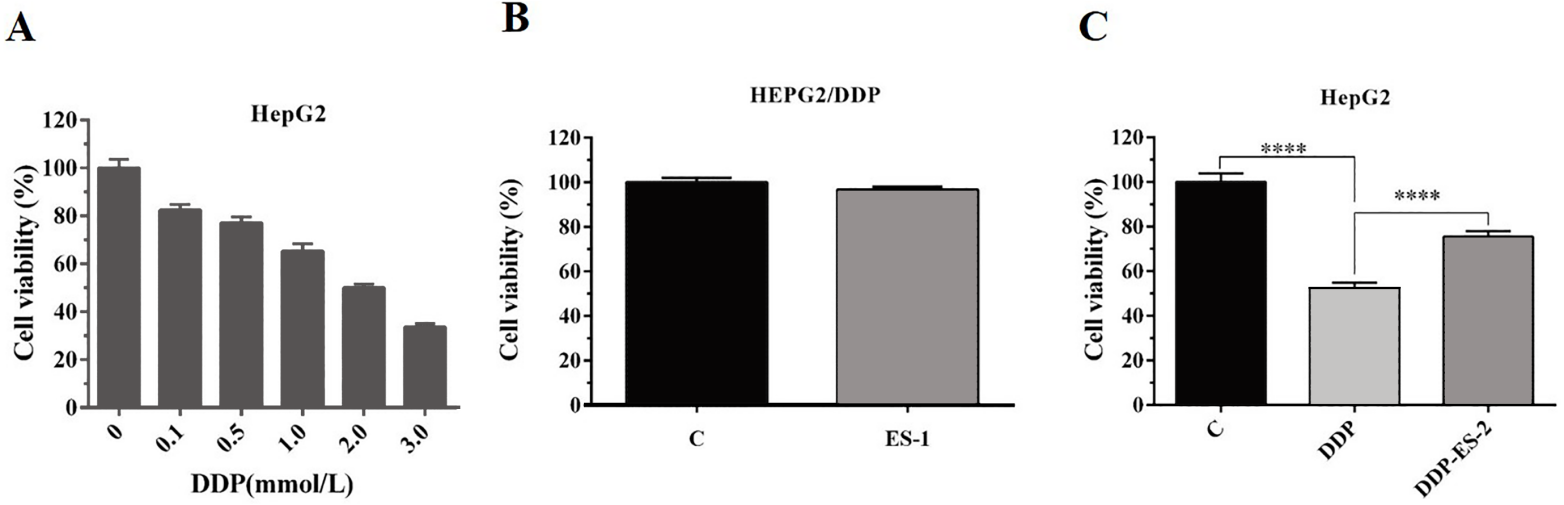
HepG2/DDP cell-derived exosomes confer a cisplatin–resistant property by mediating the HepG2 cells viability in vitro. (A) Cell cytotoxicity of DDP in HepG2 cells by MTT assay (IC50 of DDP). (B) Cell viability of HepG2/DDP cells treated with ES-1 by MTT assay. (C) Cell viability of HepG2 cells treated with DDP or DDP + ES-2 by MTT assay (^∗∗∗^*p* < 0.0001).

To analyze whether HepG2/DDP cell-derived exosomes conferred a malignant phenotype on cisplatin-sensitive cancer cells, HepG2 cells were treated with DDP or DDP + ES-2, and HepG2 DDP-resistant cell line (HepG2/DDP)were treated with ES-1. Before that, the survival rate of HepG2/DDP cells was established and we found that IC50 of DDP was about 2 mM (Fig. 2A). Then, cell viability and intracellular ROS formation were measured. Cell viability of HepG2/DDP cells treated with ES-1 was almost consistent with the control group, it means that the ES-1 treatment does not significantly affect the tolerance of HepG2 DDP cells (Fig. 2B). On the other hand, cell viability of HepG2 cells treated with DDP or DDP + ES-2 was greatly reduced compared with the control, while the viability of HepG2 cells treated with DDP + ES-2 higher than that of DDP treatment (Fig. 2C). Therefore, we hypothesized that the supplementation of ES-2 reduced the severity of DDP reaction in HCC cells.

**Figure 3:**
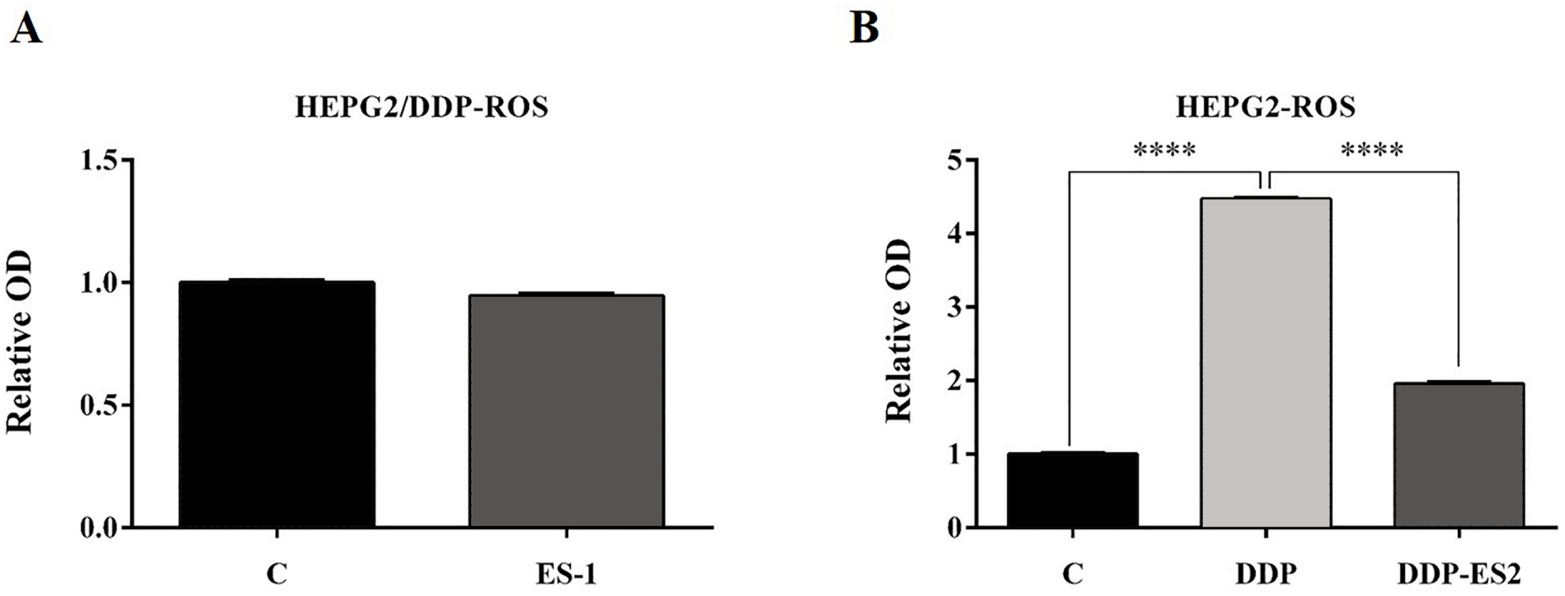
HepG2/DDP cell-derived exosomes confer a cisplatin-resistant property through ROS formation in HepG2 cells in vitro. (A) Intercellular ROS level showed no significant difference between HepG2/DDP cells treated with and without ES-1. (B) ROS formation showed a significant difference between HepG2 cells treated with DDP and DDP + ES-2 (^∗∗∗^*p* < 0.0001).

To further investigate the ES-2 mechanism of action, we applied a ROS measurement experiment. Here, intracellular ROS levels of the treated HepG2 cells and HepG2/DDP cells were quantified using a ROS assay kit. For HepG2/DDP cells, there was no significant difference in intracellular ROS level between ES-1 and the control group (Fig. 3A). But in HepG2 cells, intracellular ROS level was extremely elevated in the HepG2 cells treated with DDP compared with the control. Although the intracellular ROS level was also enhanced in the HepG2 cells treated with DDP + ES2, its concentration appeared much smaller compared with that of DDP treatment group (Fig. 3B). These results suggested that HepG2/DDP cell-derived exosomes transfer cisplatin-resistance to cisplatin-sensitive HepG2 cells through cell proliferation ability and intracellular ROS formation.

### HepG2/DDP cell-derived exosomes enhance the protein expression of P-gp in HepG2 cells

**Figure 4:**
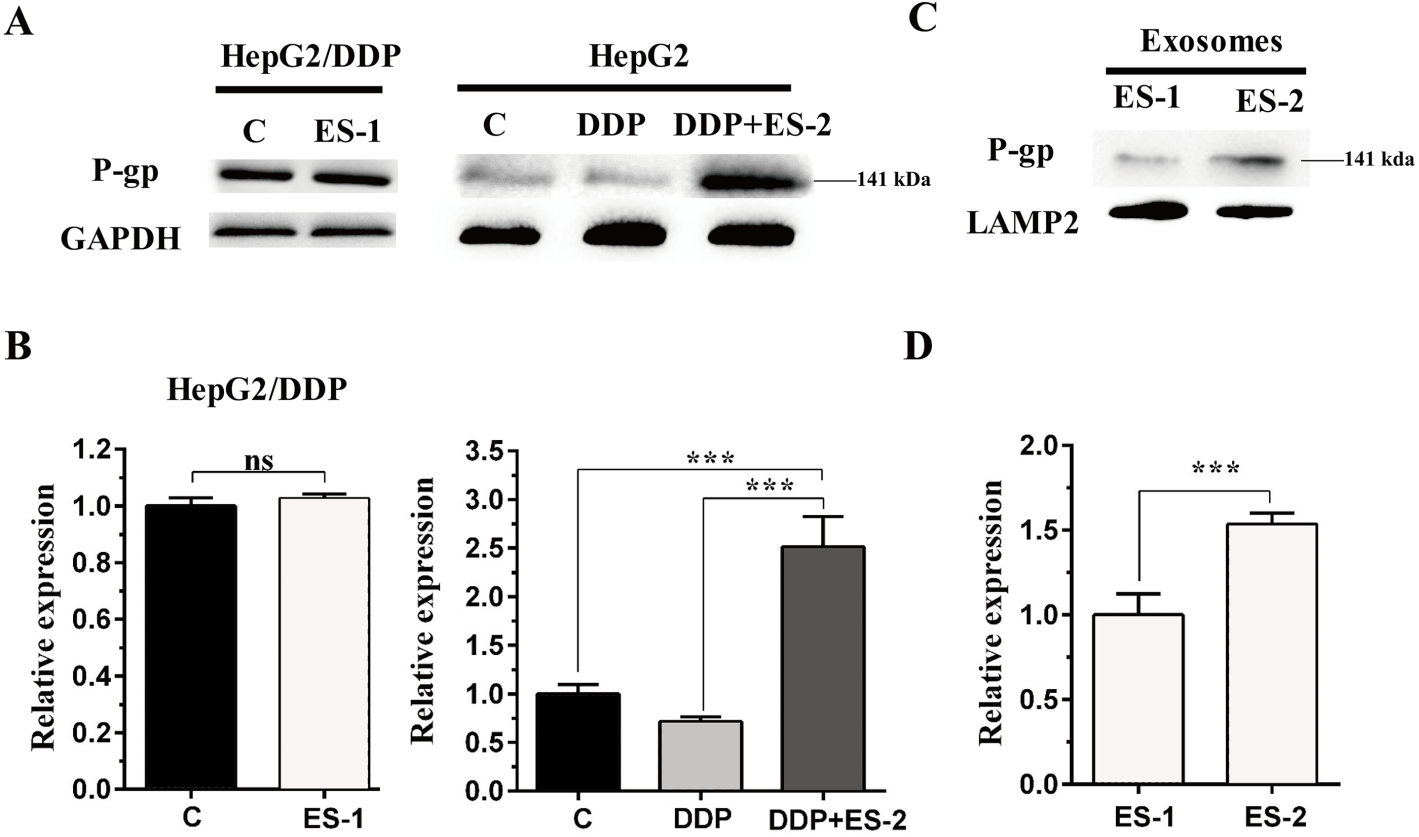
HepG2/DDP cell-derived exosomes enhance the protein expression of P-gp in HepG2 cells. (A) Western blot analysis showed the relative expression of P-gp in HepG2/DDP cells treated with ES-1 or not, and HepG2 cells treated with DDP or DDP + ES-2. (B) The gray value of (A). (C) Western blot analysis showed the relative expression of P-gp on the surface of ES-1 and ES-2. (D) The gray value of (C) ( ^∗∗∗^*p* < 0.001).

To evaluate the expression of P-gp in the HepG2 or HepG2/DDP cells, HepG2/DDP cells were treated with ES-1 and HepG2 cells were treated with DDP or DDP + ES-2. Then western blot was conducted to evaluate the protein level of P-gp in both HepG2 and HepG2/DDP cells. As shown in Fig. 4A and Fig. 4B, the protein expression of P-gp in the HepG2/DDP cells treated with ES-1 was almost consistent with that of untreated HepG2/DDP cells and greatly upregulated in HepG2 cells treated with DDP + ES-2 compared with those treated with DDP which displayed a lower expression than the controlling group. Besides, the expression of P-gp at the protein level in the ES-1 and ES-2 was confirmed and the results showed that P-gp of ES-2 on the surface was hugely enhanced compared with that of ES-1 (Fig. 4C and Fig. 4D). These results indicated that HepG2/DDP cell-derived exosomes enhance HepG2 cell’s resistance to DDP through the P-gp pathway.

### HepG2/DDP cell-derived exosomes induce a cisplatin-resistant phenotype in SMMC-7721 and Huh7 cells

**Figure 5:**
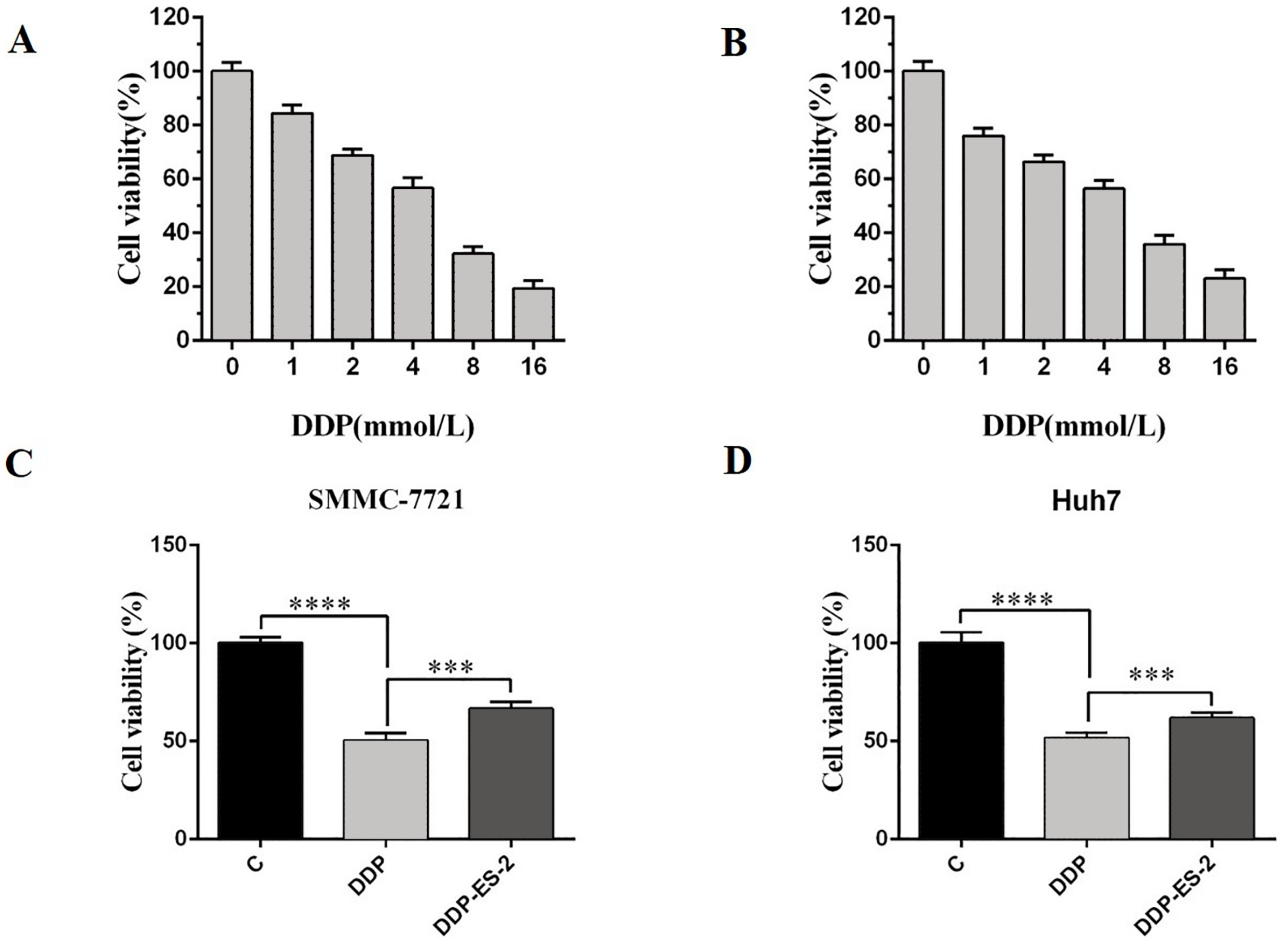
HepG2/DDP cell-derived exosomes induce a cisplatin- resistant phenotype in SMMC-7721 and Huh7 cells through cell viability. (A) Cell cytotoxicity of DDP for SMMC-7721 cells by MTT assay. (B) Cell cytotoxicity of DDP for Huh7 cells by MTT assay. (C) Cell viability of SMMC-7721 cells treated with DDP or DDP + ES-2. (D) Cell viability of Huh7 cells treated with DDP or DDP + ES-2 ( ^∗∗∗^*p* < 0.001, ^∗∗∗∗^*p* < 0.0001).

Based on the research results, we wanted to study whether the exosomes extracted from HepG2/DDP cells could have the same effect on SMMC-7721 and Huh7 cells. SMMC-7721 or Huh7 cells were treated with DDP or DDP + ES-2, and vehicle treatment was as a control. Before that, IC50 of DDP for SMMC-7721 and Huh7 cells was determined, showing 4.935 mM (Fig. 5A) and 4.493 mM (Fig. 5B), respectively. For cell viability assay, we found that cell viability of SMMC-7721 cells treated with DDP was greatly reduced compared with the control, while cell viability of SMMC-7721 cells treated with DDP + ES-2 was less reduced compared with that of DDP treatment group (Fig. 5C). Accordingly, the cell viability of Huh7 cells was consistent with that of SMMC-7721 cells (Fig. 5D).

**Figure 6:**
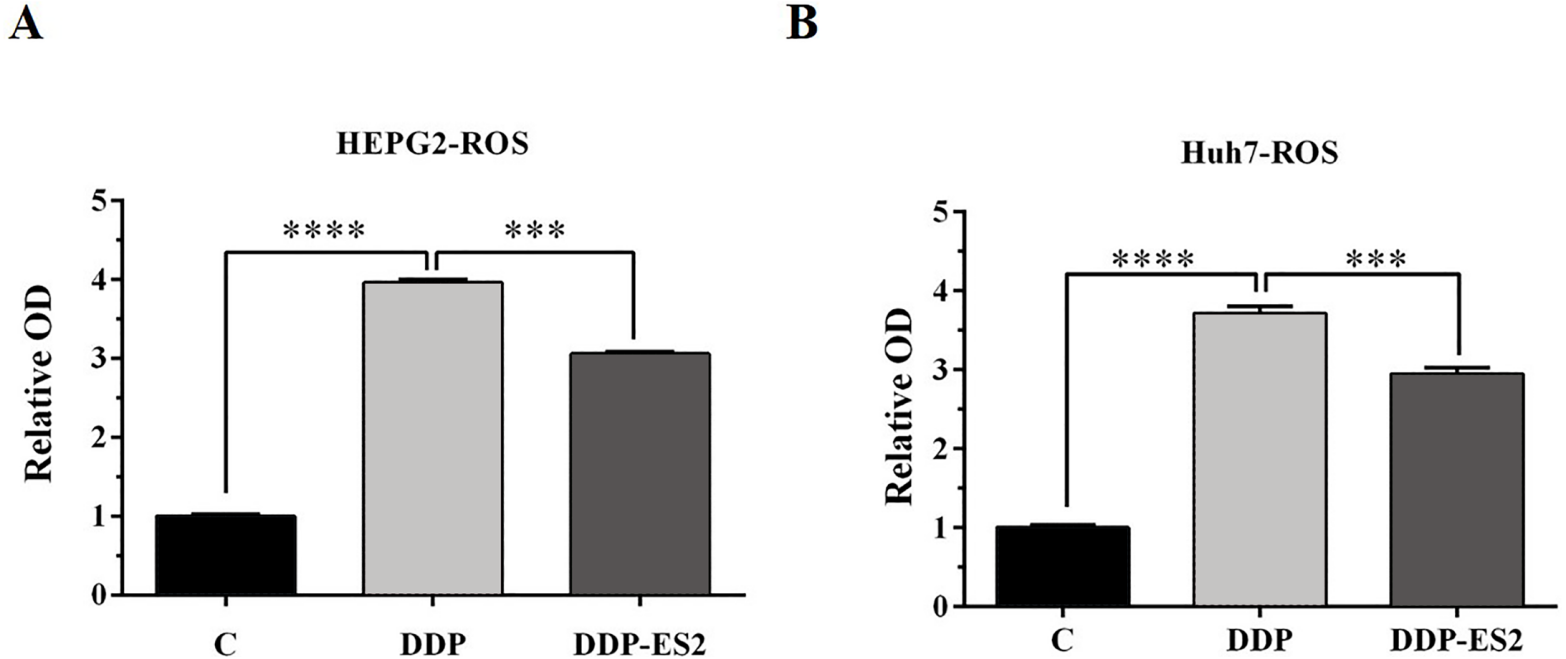
HepG2/DDP cell-derived exosomes induce a cisplatin- resistant phenotype in SMMC-7721 and Huh7 cells through ROS formation. (A) ROS formation showed a significant difference between DDP and DDP + ES-2 treatment for SMMC-7721 cells. (B) ROS formation showed a significant difference between DDP and DDP + ES-2 treatment for Huh7 cells ( ^∗∗∗^*p* < 0.001, ^∗∗∗∗^*p* < 0.0001).

Moreover, the intracellular ROS of treated SMMC-7721 cells was measured, and as depicted by Fig. 6A, the ROS level of SMMC-7721 cells treated with DDP was dramatically improved compared with that of control. Also, the ROS formation level of SMMC-7721 cells treated with DDP + ES-2 was significantly reduced compared with that of DDP treatment group. Notably, the trend of results of cell viability and ROS level of Huh7 cells treated with DDP + ES-2 was similar to that ofSMMC-7721 cells (Fig. 6B), suggesting that ES-2 transferred cisplatin-resistance to SMMC-7721 and Huh7 cells through enhancing cell proliferation and intracellular ROS formation.

### HepG2/DDP cell-derived exosomes upregulate the expression of P-gp in SMMC-7721 and Huh7 cells

**Figure 7:**
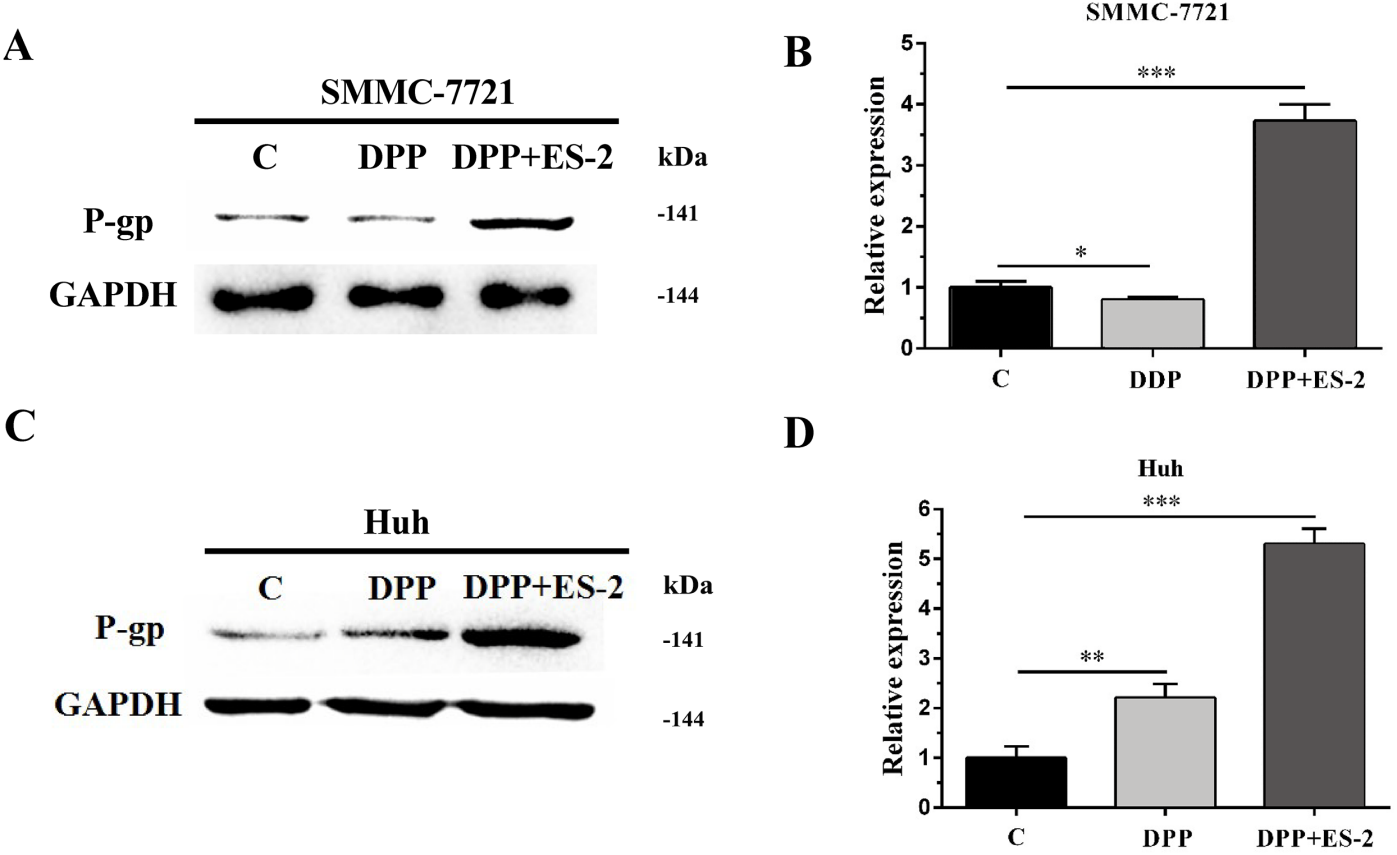
HepG2/DDP cell-derived exosomes upregulate the expression of P-gp in SMMC-7721 and Huh7 cells. (A) Western blot analysis showed the relative expression of P-gp between DDP and DDP + ES-2 treatment for SMMC-7721 cells. (B) The gray value of (A). (C) Western blot analysis showed the relative expression of P-gp between DDP and DDP + ES-2 treatment for Huh7 cells. (D) The gray value of (C) ( ^∗^*p* < 0.05, ^∗∗^ *p* < 0.01, ^∗∗∗^ *p* < 0.001).

Similarly, expression of the P-gp protein in SMMC-7721 cells treated with DDP or DDP + ES-2 was evaluated via WB. The results revealed a transient upregulated protein expression of P-gp in the DDP + ES-2, and a small decrease in the DDP group compared with the controlling group (Fig. 7A and Fig. 7B). Curiously, Huh7 cells treated with DDP + ES-2 displayed a similar trend of result with that in SMMC-7721 cells. On contrary, in comparison with the control group, the protein expression of P-gp was lightly increased in Huh7 cells treated with DDP. (Fig. 7C and Fig. 7D). These outcomes clarified that HepG2/DDP cell-derived exosomes increased the protein expression of P-gp in various HCC cells, although the DDP could affect those cells differently.

## Discussion

Cisplatin (DDP), is reputed for its utilization in cancer treatment, and has shown fair efficacy in HCC handling; it is widely used as a chemotherapy agent for advanced HCC patients (Lencioni et al., 2016). However, the DDP usage is still disqualified as a first-line treatment due to its deepening drug resistance that limits the clinical efficacy of DDP therapy for HCC patients (Attwa & El-Etreby, 2015). It was reported that DDP resistance could be associated with DNA damage in interaction with mitochondria, lysosomes or other organelles, reducing the sensitivity of DDP to tumor cells (Chauhan et al., 2003). Recently, Sharma (2017) clarified that exosomes from drug-resistant cancer cells could transfer chemoresistance to chemo-sensitive cancer cells. Whether exosomes from DDP-resistant HepG2 cells transmit DDP-resistance to other HCC-sensitive cells remains unknown. Thus, a DDP-resistant HepG2 cell (HepG2/DDP) was produced from its parent cell and its exosomes were isolated. Therefore, verifying that exosomes from HepG2/DDP transfer chemoresistance to HCC cells constituted the aim of the present study.

A growing number of studies have evidenced that chemo-resistant tumor cells can release exosomes that transmit drug resistance during tumorigenesis. Kuan Ning et al. proposed that UCH-L1-containing exosomes can transfer chemoresistance to recipient cells in breast cancer (Ning et al., 2017). In addition, exosomes derived from PTX-treated with aggressive cell MDAMB231 have been suggested to enhance chemoresistance (Kreger et al., 2016). To analyze whether HepG2/DDP cell-derived exosomes might confer a malignant phenotype on paclitaxel-sensitive cancer cells, exosomes from HepG2 and HepG2/DDP cell were separated with a typical round shape and verified with their corresponding surface markers including CD9, CD63 and CD81. All three surface markers were showed to be positively expressed in both ES-1 and ES-2 justifying successful isolation of exosomes (Fig. 1A). To further investigate whether exosomes were responsible for the spread of chemoresistance to other sensitive cancer cells, HepG2 cells were treated with ES-2 and HepG2/DDP cells were treated with ES-1, and we found that cell viability and intercellular ROS barely changed in the HepG2/DDP treated with ES-1compared with control while cell viability was enhanced and intercellular ROS formation was reduced compared with sensitive cells treated with DDP (Fig. 2C). These data suggested that the induction of a cisplatin-resistant phenotype in HepG2 cells could be assigned to exosomes derived from HepG2/DDP resistant cells. To validate the above hypothesis, the protein expression of P-gp was further purchased. As expected, the protein expression of P-gp in HepG2/DDP cells treated with ES-1 was almost consistent with that of control while the protein expression of P-gp was significantly upregulated in the HepG2 cells treated with DDP + ES-2 (Fig. 4A and Fig. 4B). It is important to notice that protein expressions of P-gp from ES-1 and ES-2 were also assessed into HepG2 cells, and we found that P-gp proteins were more expressed in ES-2 than in ES-1 (Fig. 4C and Fig. 4D). These results confirmed that the recipient chemo-sensitive HepG2 cells acquired the chemo-resistant phenotype through the ES-2 transfer.

Moreover, to elucidate whether the ES-2 could transfer chemo-resistant phenotype to other HCC cells, SMMC-7721 and Huh7 cells were selected for further verifications. The IC50 of DDP from SMMC-7721 and Huh7 cells were almost similar, exhibiting 4.935 mM and 4.493 mM, respectively. SMMC-7721 or Huh7 cells were therefore subjected to the same treatments as for the sensitive HepG2 cells. The cell viability of SMMC-7721 cells treated with DDP + ES-2 was less reduced compared with that of the DDP treatment group; accordingly, the cell viability of Huh7 cells was consistent with that of SMMC-7721 cells (Fig. 5C and Fig. 5D). This result confirms that ES-2 can transfer DDP drug resistance to various HCC cells by promoting their cell viability. The increase of intracellular ROS formation in SMMC-7721 or Huh7 cells treated with the reduction observed in the same cells under the DDP + ES-2 was significant (Fig. 6). A recent study has demonstrated that the ROS generation in mitochondria was stimulated to defeat various cancer drug tolerances (Wang et al., 2018). Here, the augmentation of ROS production in HCC cells treated with DDP confirms the drug resistance toward the cisplatin DDP while the ROS production slowed down when DDP is supplied with ES-2. This means that the ES-2 lightens the drug resistance in HCC sensitive cells. Similarly, the protein expression of P-gp in SMMC-7721 or Huh7 cells treated with DDP and DDP + ES-2 showed upregulated protein expression of P-gp in the DDP + ES-2 while exhibiting a small decrease in the DDP group compared with the controlling group. Although this section proved that the ES-2 conferred greater protein expression to P-gp in HCC sensitive cells, the protein expression of P-gp could vary from one HCC cell to another (Fig. 7).

## Conclusion

To sum up, the present recherche article demonstrated that exosomes from drug-resistant cell HepG2/DDP could transfer the chemoresistance property to the sensitive cells in the HCC by mediating the intracellular ROS production and the P-gp protein activities respectively. Thus, our results provide considerable guidance in support to the chemotherapy of HCC nursing with the clinical cisplatin DDP.

## Additional Information and Declarations

### Data Availability

The following information was supplied regarding data availability: Data are available at Figshare: qin (2020): raw data. figshare. Dataset. https://doi.org/10.6084/m9.figshare.13442978.v1
